# MRI-guided coupling for a focused ultrasound system using a top-to-bottom propagation

**DOI:** 10.1186/s40349-017-0087-x

**Published:** 2017-01-23

**Authors:** Marinos Yiannakou, George Menikou, Christos Yiallouras, Christakis Damianou

**Affiliations:** 1 Cyprus University of Technology, Limassol, Cyprus; 2 City, University of London, London, UK; 3 MEDSONIC LTD, Limassol, Cyprus

**Keywords:** Ultrasound, Robot, MRI, Fibroid, Cancer

## Abstract

**Background:**

A novel magnetic resonance imaging (MRI)-conditional coupling system was developed that accommodates a focused ultrasound (FUS) transducer. With this coupling system, the transducer can access targets from top to bottom. The intended clinical application is treatment of fibroids using FUS with the patient placed in supine position.

**Methods:**

The coupling system was manufactured using a rapid prototyping device using *acrylonitrile butadiene styrene* (ABS) plastic. Coupling to a gel phantom was achieved using a water bag filled with degassed water. The FUS transducer was immersed in the water bag.

**Results:**

The coupling system was successfully tested for MRI compatibility using fast-gradient pulse sequences in a gel phantom. The robotic system with its new coupling system was evaluated for its functionality for creating discrete and multiple (overlapping) lesions in the gel phantom.

**Conclusions:**

An MRI-conditional FUS coupling system integrated with an existing robotic system was developed that has the potential to create thermal lesions in targets using a top-to-bottom approach. This system has the potential to treat fibroid tumors with the patient lying in supine position.

## Background

The available main treatment options for uterine fibroids include hysterectomy, myomectomy, and uterine artery embolization. Hysterectomy is the primary option for resolving fibroid-associated symptoms. Uterine artery embolization (UAE) is major treatment option for fibroids which was introduced in 1995 [1]. This treatment option involves femoral artery catheterization and intra-arterial infusion of embolization particles. As a result, UAE produces ischemia of the fibroid uterus, thus reducing significantly the volume of fibroids [1]. Another treatment option for uterine fibroids involves hormonal manipulation. Gonadotrophin-releasing hormone (GnRH) is predominantly used for the temporary reduction of fibroid volume by as much as 60% sometimes [2].

Another treatment option which is not widely accepted is cryoablation [3]. With cryoablation, the uterine fibroid is cooled to very low temperatures. This option is introduced using either laparoscopic or hysteroscopic access [4, 5].

Another option deployed recently is magnetic resonance imaging-guided focused ultrasound (MRgFUS) [6]. MRgFUS is an effective and completely noninvasive modality. MRgFUS may be used as a fertility-preserving option for some cases. The first treatment of uterine fibroids using MRgFUS was performed in 2003 and was implemented by Stewart and colleagues [6]. The results of this study were promising, and thus lead to additional clinical trials. The goal of the additional trials was to evaluate the efficacy of MRgFUS in larger number of patients. These studies showed significant reduction of clinical symptoms. Additionally, improvement in life quality was reported at 6, 12, and 24 months. Until now, more than 8500 patients have been treated with MRgFUS worldwide. Only few side effects were reported [7]. The acceptance rates from patients are evaluated as high [7]. In recent reports of clinical trials, with proper selection of patient population, the reduction in volume is often more than 50% [8]. However, despite the good clinical results, one of the disadvantages of this procedure is the lengthy procedure time (i.e., low time efficiency) which is a big problem when treating large fibroids.

The first MRgFUS commercial system (Exablate) to be used for the treatment of fibroids was developed by InSightec (Tirat Carmel, Israel) [9]. The system uses a phased-array transducer operating close to 1 MHz. The transducer is positioned close to the target using a robotic system. The whole system is placed in the magnetic resonance imaging (MRI) table. Treatment is performed with the patient in the prone position and under light sedation, with active monitoring of vital signs.

In 2009, Philips developed the MRgFUS robotic system (Sonalleve, Philips Healthcare) that was Conformité Européenne (CE)-marked for the treatment of uterine fibroids [10, 11]. Treatments were performed using a phased-array 256-channel transducer (radius of curvature 12 cm, aperture 13 cm; operable at 1.2 MHz) equipped with a mechanical displacement device with 5 degrees of freedom (three linear and two angular). This system is able to perform volumetric ablation. With this system, the patient is also placed in prone position.

This study includes the conversion of a robotic system intended for brain applications to a robotic system that can be used for accessing fibroids. This is achieved by modifying the coupling system, thus allowing top-to-bottom coupling (axial in MRI). The system was evaluated in a gel phantom for producing discrete and overlapping lesions. The system uses a single-element transducer, which makes the system less complex and cost-effective compared to systems that use phased-array technology.

## Methods

### Coupling for fibroids

**Fig. 1:**
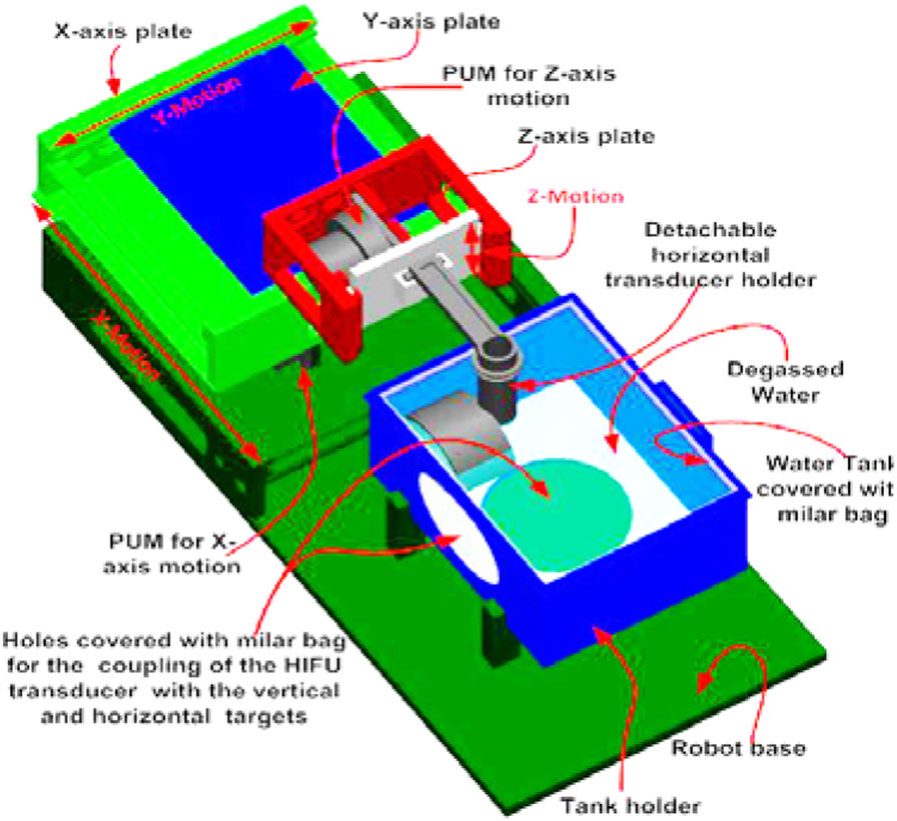
The existing robotic system dedicated for the brain

**Fig. 2:**
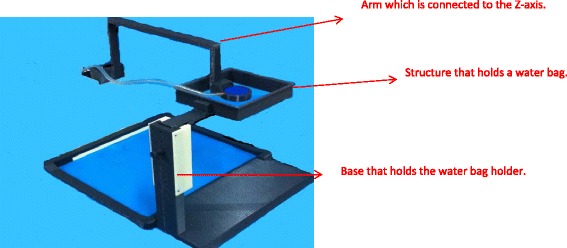
The developed coupling that can be used for a top-to-bottom access of ultrasound to targets

**Fig. 3:**
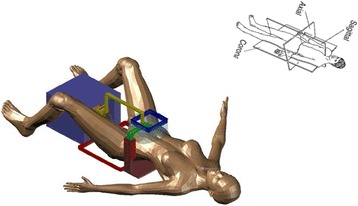
The concept of using the modified robotic system for access to the fibroids

An existing positioning device with three axes (*X*, *Y*, *Z*) was used [12]. Figure 1 shows the existing robotic system dedicated for the brain. The coupling system was modified so that top-to-bottom access was possible. A modified arm was developed that was inserted in a coupling structure that makes coupling to fibroids (in this study, gel phantom). The three axes were driven by piezoelectric ultrasonic motors (USR60-S3N, Shinsei Kogyo Corp., Tokyo, Japan). Optical encoders were used (US Digital Corporation, Vancouver, WA 98684, USA). The encoder output was connected to the counter input of a data acquisition board USB 6251 (NI, Austin, USA). Figure 2 shows the developed coupling that can be used for a top-to-bottom access of ultrasound to targets. This prototype coupling system includes a transducer arm which is connected to the *Z*-axis, a holder for the water bag, and a base that holds the water bag holder. This coupling system is manually positioned to the patient. This structure includes a movable water bag (steps of 1 cm), a transducer holder, and an arm that is fixed to the existing robot. Figure 3 shows the concept of using the modified robotic system for access to the fibroids.

### Experimental setup

The arm of the robotic system with the 1-MHz transducer was immersed inside the water bag. The water bag was filled with degassed water. The transducer was placed above the gel phantom. The distance of the transducer from the phantom was such that the beam focus was placed in the middle of the gel phantom. The phantom was wrapped around by the GPFLEX coil (USA instruments, Cleveland, OH, USA) to perform all the imaging studies.

### FUS system

The effectiveness of the system was evaluated by creating lesions in polyacrylamide gel phantom (ONDA Corporation, Sunnyvale, CA, USA). The FUS system consists of an RF amplifier (RFG 750W, JJA instruments, Seattle, WA, USA) and a spherical transducer made from piezoelectric ceramic (Sonic Concepts, USA). The transducer operates at 1.14 MHz and has focal length of 10 cm and diameter of 3 cm. The acoustical power of 20 W was applied in continuous mode for 60 s. With 20 W/60 s, the goal was to get temperature maps and test the MR thermometry without damaging the gel permanently. In another exposure that creates lesions, the power used was 30 W for 30 s. With this transducer, and focal depth for 30 W, lesions are created with a 20-s exposure or higher. The heating of the system was evaluated in the gel phantom. Degassed water was placed between the transducer, water bag, and the gel phantom, thus providing good acoustical coupling between the gel phantom and the FUS transducer. The attenuation of the gel as reported by the manufacturer was 0.6 dB/cm at 1 MHz [13].

### MR imaging

The robotic FUS system was tested in a 1.5-T MR system (Signa, General Electric, Fairfield, CT, USA) using a lumbar spine coil (USA instruments, Cleveland, OH, USA).

### MR thermometry

The temperature elevation during FUS exposures was estimated using the proton resonance frequency (PRFS) shift method [14]. This method relates the phase shift derived from the frequency shift of the MR signal due to the local temperature elevation (Δ*T*). This relationship is described by the following:

1 $$ \varDelta T=\frac{\varphi (T)-\varphi \left({T}_0\right)}{\gamma \alpha {B}_0\mathrm{T}\mathrm{E}}, $$

where φ(*T*) and φ(*T*_0_) are the absolute phases of the MR signal at a starting and final temperature *T* and *T*_0_, respectively; *γ* is the gyromagnetic ratio; *α* is the PRF change coefficient (0.01 ppm/°C); *B*_0_ is the magnetic field strength; and TE is the echo time.

**Fig. 4:**
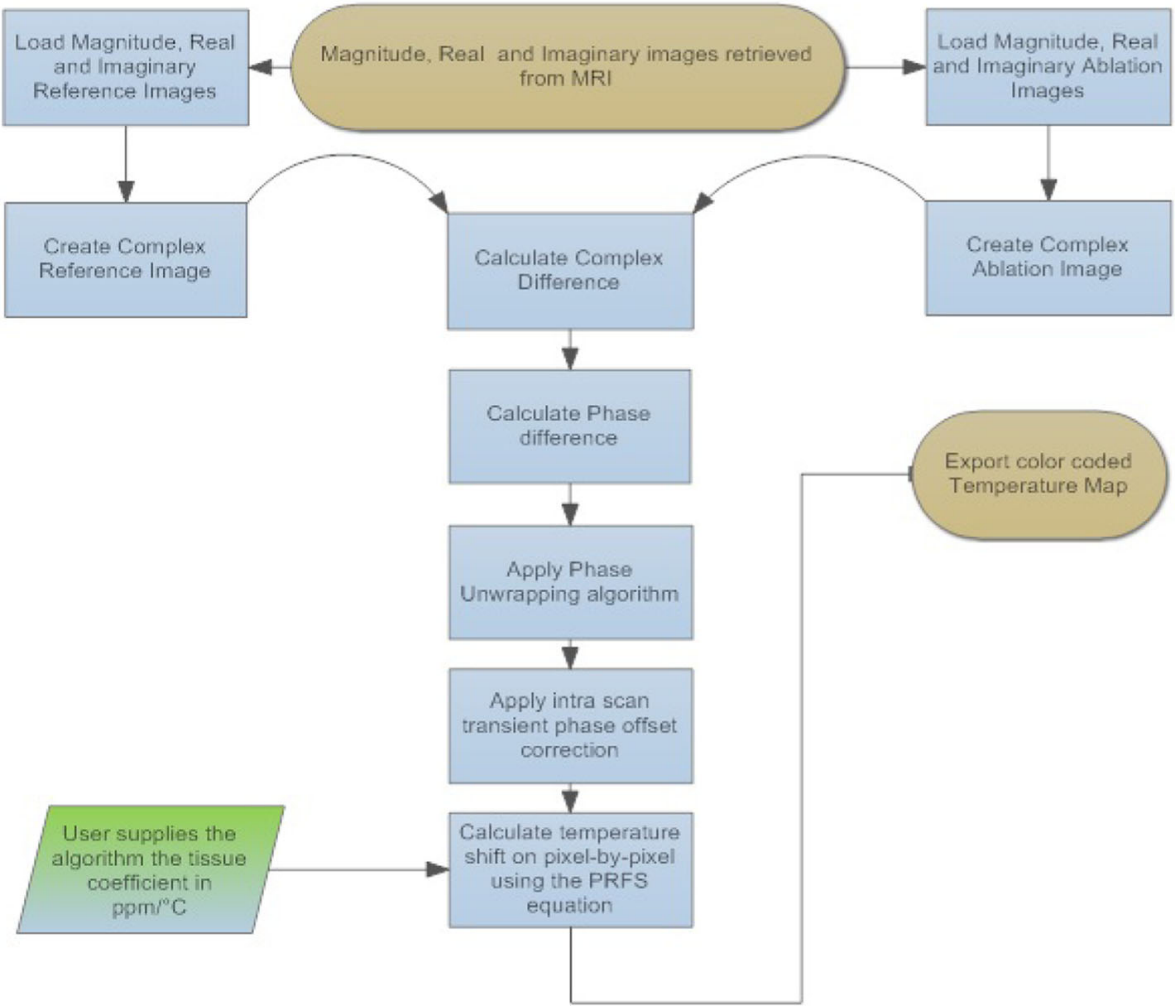
Software flowchart for estimating MR thermometry

The spoiled gradient echo sequence (SPGR) was used for thermometry: repetition time (TR) 38.5 ms, TE 20 ms, bandwidth (BW) 15 kHz, matrix 128 × 128, slice thickness: 10 mm, and number of excitations (NEX): 1. The temporal resolution of thermometry was about 12 s. Phase maps were reconstructed by calculating the phase on a pixel-by-pixel basis after combining pixel data from real and imaginary channels. Although the scanner was capable of producing directly phase image reconstructions, the applied intra-scan gradient non-linearity corrections induce phase interpolation problems. All of the image processing was performed with custom-made software developed in MATLAB (MathWorks, Natick, USA). Temperature color-coded maps were produced by adjusting the color map (blue to red) for a range of minimum to maximum region of interest (ROI) temperature. Figure 4 shows the flowchart of the software that estimates temperature using the PRFS method.

High-resolution MR imaging was performed to visualize the FUS lesions in the gel phantom using T2-weighted fast spin echo (FSE) sequence (imaging parameters, TR 2500 ms, TE 60 ms, slice thickness 3 mm, matrix 256 × 256, field of view (FOV) 16 cm, NEX 3, and echo train length (ETL) 8).

## Results

**Fig. 5:**
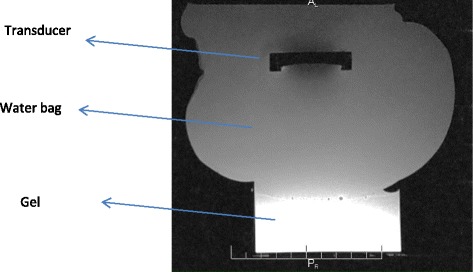
MR image using T2-W FSE of the coupling to the gel of the robotic system

Figure 5 shows the MR image using T2-weighted (T2-W) FSE of the coupling to the gel of the robotic system. In this image, the transducer-water bag-gel phantom arrangement is shown demonstrating the excellent coupling to the gel phantom.

**Fig. 6:**
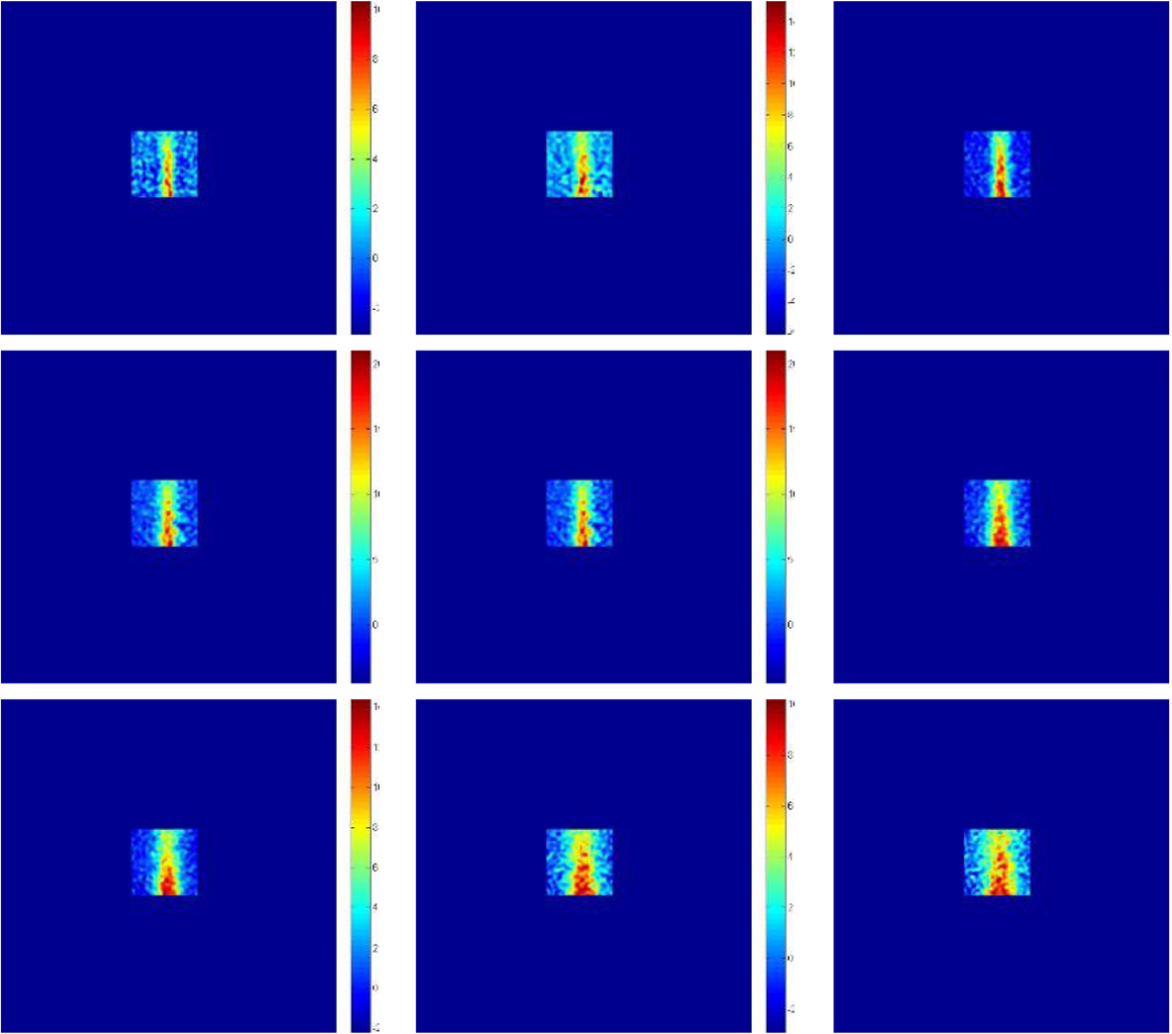
Axial temperature maps produced by this transducer in a plane perpendicular to the face of the transducer. The acoustical power of 20 W was applied for 60 s

**Fig. 7:**
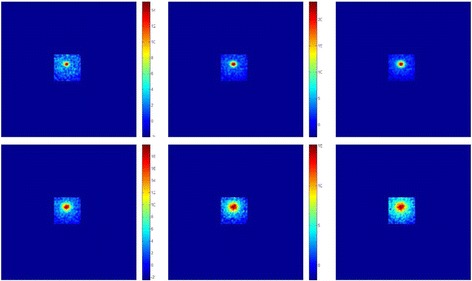
Coronal temperature maps produced by this transducer in a plane parallel to the face of the transducer. The acoustical power of 20 W was applied for 60 s

Figure 6 shows the axial temperature maps produced by this transducer. The acoustical power of 20 W was applied for 60 s. During the first five images, the FUS transducer was activated. Having observed the focal beam in a plane perpendicular to the transducer face, the next step was to evaluate the temperature maps in a plane parallel to the transducer face. Figure *7* shows the coronal temperature maps produced by this transducer. The acoustical power of 20 W was applied for 60 s. During the first five images, FUS was activated.

**Fig. 8:**
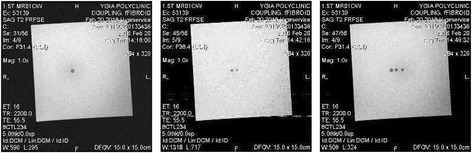
MR images (using T2-W FSE) of three discrete thermal lesions created in the gel phantom by moving the X stage of the robotic system. The acoustical power used was 30 W for 30 s. The spatial step between lesions was 5 mm

**Fig. 9:**
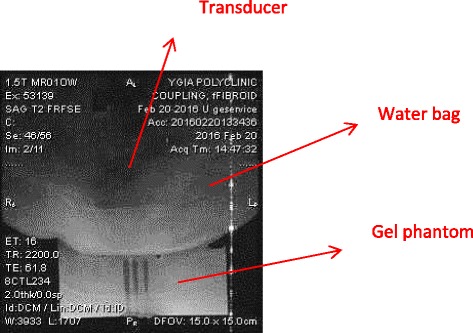
MR image (using T2-W FSE) of the three discrete thermal lesions of Fig. 7 in axial plane demonstrating the penetration deep in the gel (plane perpendicular to the transducer face)

Figure 8 shows the MR images (using T2-W FSE) of three discrete thermal lesions created in the gel phantom by moving the X stage of the robotic system. The acoustical power used was 30 W for 30 s. The spatial step between lesions was 5 mm. With the transducer and focal depth with a 30 W and 30 s exposure, the lesion width is 3.3 mm, and the lesion length is 24 mm (maximum temperature recorded was 82 °C). With 60 s exposure and 30 W, the lesion width is 4.2 mm and the lesion length is 28.4 mm (maximum temperature recorded was 94 °C). At higher power, the lesion size does not change much since conduction carries the heat away. Also, a higher power will cause the temperature to exceed 100 °C (tissue boiling point). Figure 9 shows the MR image (using T2-W FSE) of the three discrete thermal lesions of Fig. 8 in axial plane demonstrating the penetration deep in the gel (plane perpendicular to the transducer face).

**Fig. 10:**
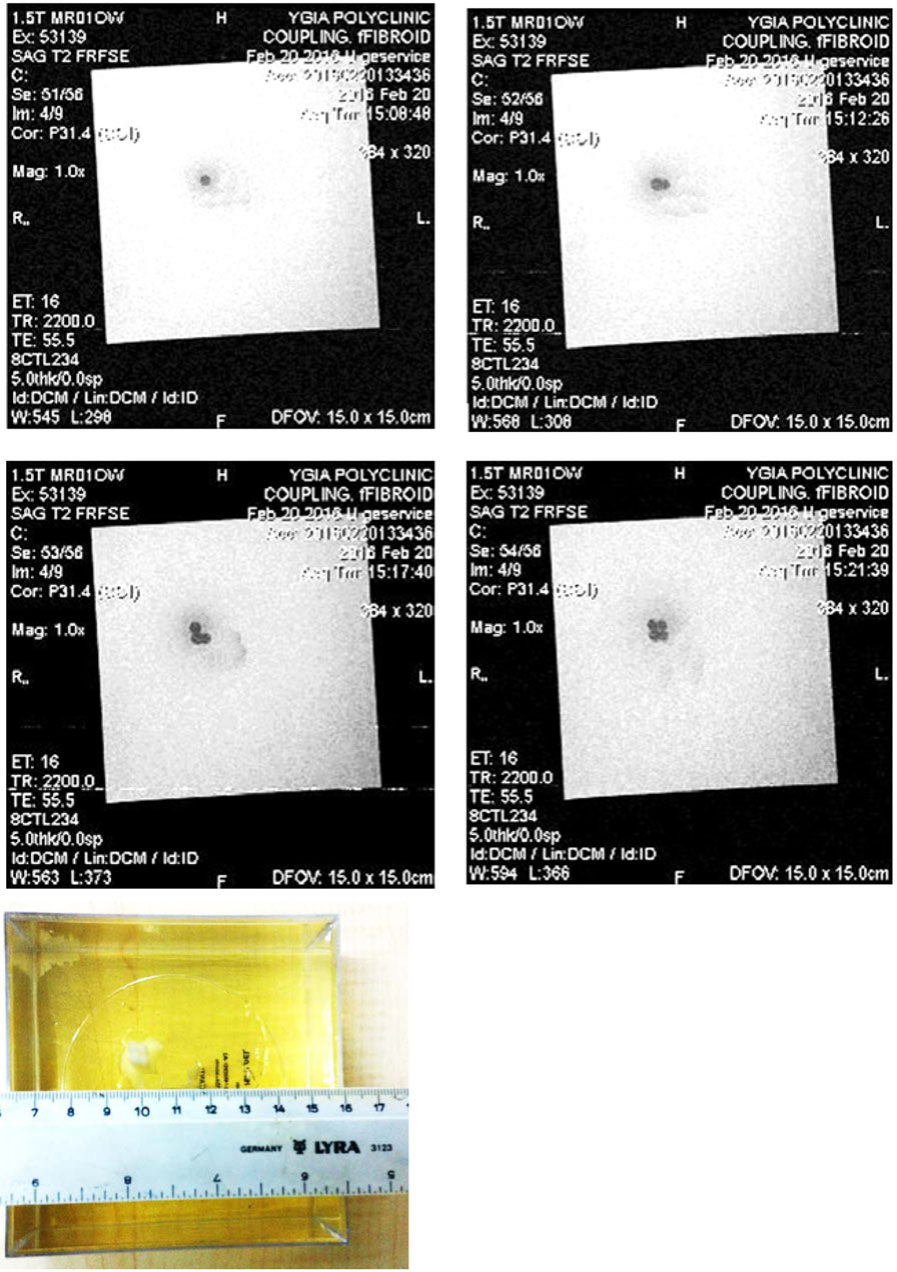
MR images (using T2-W FSE) of four overlapping thermal lesions created in the gel phantom by moving the X and Y stages of the robotic system in a 2 × 2 square grid. The acoustical power used was 30 W for 30 s. The spatial step between lesions was 3 mm

Figure 10 shows the MR images (using T2-W FSE) of four overlapping thermal lesions created in the gel phantom by moving the X and Y stages of the robotic system in a 2 × 2 square grid. The acoustical power used was 30 W for 30 s. The spatial step between lesions was 3 mm. Because the width of the lesion is close to 3 mm (see Fig. 8), then to get overlapping lesions, the step size had to be 3 mm. This figure clearly demonstrates the effectiveness of the positioning device for creating large lesions for the purpose of thermal ablation.

## Discussion

Our study was inspired by several clinical trials that have shown that MRgFUS destroys fibroids [6, 7]. In these studies, the FUS transducer is placed in a water container which is integrated in the MRI table. The coupling with this technology is bottom to top, and the patient sits lying on the table in the prone position. The focal beam with the existing technologies is moved using phased-array technology which is very complicated and expensive. In this study, we presented an alternative technology of MRgFUS which is based on mechanical movement of a single-element spherically focused transducer. The proposed technology is simpler and cost-effective. The coupling to the target is top to bottom, and the patient may sit in supine position on the table. The major challenge of this technology is the coupling of the transducer to the target. This challenge has already been solved by several studies. The team of Theraclion “makes” a contact to the thyroid or to the breast [15] with a transducer placed on the top of the target reporting a more comfortable patient placement. This same concept was shown in another study performed by our group [16].

The major advantage of the proposed robot is that the patient can be placed on the MRI table in supine position. In the current systems, the patients are placed in the prone position. Since sometimes the treatment procedure can be long, lasting up to 3 hours per session [17, 18], the proposed system could provide better comfort for the patients. Additionally, the proposed system can access multiple anatomical locations.

Based on the American Society for Testing and Materials (ASTM) document F2503 described by Stoianovici et al. [19], the proposed system is classified as MRI-conditional because of the use of the FUS transducer, piezoelectric motors, and optical encoders. The piezoelectric motors, the transducer, and the optical encoders require the use of electricity and therefore, the system is MRI-conditional. Pneumatic systems [19] on the other hand are classified as MRI safe, because no electricity is used.

## Conclusions

The proposed technology is a continuation of other MRgFUS technologies designed by our group for various applications. Such system were developed for brain ablation using three Cartesian axes [12], prostate ablation using one linear and one angular axis [20, 21], and gynecological tumor ablation using one linear and one angular axis [22].

The FUS system produced lesions in gels successfully. The length and width of these lesions can be easily controlled by varying the intensity and time of exposure. These discrete and overlapped lesions were produced using the *X* and *Y* axes. The appearance of these lesions was demonstrated using MRI and proved that the linear stages moved with great accuracy. The degree of accuracy of the linear stages was also demonstrated in other articles of our group (for example, [12, 20, 21]). In future experiments, we plan to use thicker gel phantoms, in order to create lesions in a model which is closer to the size of anatomy involved for the case of fibroids.

In the future, some registration marks should be placed in the proposed system in order to transfer the position of the transducer or arm in the MRI images. Additionally, an appropriate MRI coil should be selected. Currently, the lumbar spine was used (best option available to us). Better signal can be received if a dedicated coil is placed in proximity to the target. The proposed system which is modular can be easily modified to explore other applications with a top-to-bottom coupling arrangement (for example, breast or thyroid cancer). Finally, for better maneuverability, the proposed robot can be enhanced by the addition of additional motion stages to reach the performance reported by the FUSBOT robotic system [23].

## Abbreviations

ABS: Acrylonitrile butadiene styrene
ASTM: American Society for Testing and Materials
BW: Bandwidth
CE: Conformité Européenne
ETL: Echo train length
FSE: Fast spin echo
FUS: Focused ultrasound
GnRH: Gonadotrophin-releasing hormone
MRgFUS: Magnetic resonance-guided focused ultrasound system
MRI: Magnetic resonance imaging
NEX: Number of excitations
PRFS: Proton resonance frequency
ROI: Region of interest
SPGR: Spoiled gradient echo sequence
T2-W: T2-weighted
UAE: Uterine artery embolization
